# Ponction biopsies rénales dans le Service de Néphrologie de Fès: indications et résultats: à propos de 522 cas

**DOI:** 10.11604/pamj.2016.24.21.3982

**Published:** 2016-05-06

**Authors:** Houda Mbarki, Khadija Alaoui Belghiti, Taoufiq Harmouch, Adil Najdi, Mohamed Arrayhani, Tarik Sqalli

**Affiliations:** 1 Service de Néphrologie, CHU Hassan II, Fès, Maroc; 2 Laboratoire d'Anatomopathologie, CHU Hassan II, Fès, Maroc; 3 Faculté de Médecine et de Pharmacie de Fès, Maroc; 4 Laboratoire d'Epidémiologie, de Recherche Clinique et de Santé Communautaire, Faculté de Médecine et de Pharmacie de Fès, Maroc

**Keywords:** Biopsie rénale, néphropathies, diagnostic, Renal biopsy, nephropathies, diagnosis

## Abstract

L'apport de la ponction biopsie rénale (PBR) dans le diagnostic, le choix thérapeutique et l’évaluation pronostique des néphropathies est considérable. Aucune étude marocaine n'a évalué la pratique et l'apport de la PBR. Notre objectif est d’étudier les indications de la PBR, déterminer la fréquence des maladies rénales identifiées par PBR dans notre région et de faire une confrontation entre les données clinico-biologiques et le diagnostic historique. Notre étude menée entre Janvier 2009 et Décembre 2012, est rétrospective. Nous avons inclus tous les patients du service de Néphrologie du CHU Hassan II de Fès ayant bénéficié d'une biopsie de reins natifs. 522 PBR ont été réalisées. Nous avons exclu 8 biopsies devant le manque de renseignements et avons donc retenu 514. L’âge moyen des patients au moment de la PBR est de 39 ±17 ans (3-82 ans). Le sex ratio est de 0,9. Le syndrome néphrotique est le diagnostic clinique le plus fréquent à tous les âges (58,2%). Les néphropathies glomérulaires représentent 94,2% des maladies rénales diagnostiquées, leur distribution varie selon l’âge des patients. La PBR a confirmé le premier diagnostic suspecté cliniquement dans 40,65% des cas, alors qu'elle a révélé un diagnostic inattendu chez 22,5% d'entre eux. Le diagnostic syndromique permet d'orienter vers la maladie rénale la plus probable et de guider les thérapeutiques urgentes en attendant les résultats de la PBR. Mais il ne peut en aucun remplacer la PBR qui reste le gold standard.

## Abstract (en)

The contribution of renal needle biopsy (RNB) to make a diagnosis, a treatment selection and a prognostic evaluation of nephropathies is significant. No Moroccan study has evaluated the practice and the contribution of RNB. Our aim was to study RNB indications, to determine the frequency of kidney diseases identified by RNB in our region and make a comparison between clinical and biological data and histological diagnosis. This is a retrospective study conducted between January 2009 and December 2012. We included all patients in the Department of Nephrology, CHU Hassan II, Fez, who underwent biopsy of native kidneys. 522 RNB were performed. We excluded 8 biopsies due to lack of informations and 514 were retained. The average age of the patients at the time of RNB was 39±17 years (3-82 years). Sex ratio was 0.9. Nephrotic syndrome was the most common clinical diagnosis to all ages (58.2%). Glomerular nephropathies represent 94,2% of diagnosed renal diseases, their distribution varies according to patients’ age. RNB confirmed the first clinically suspected diagnosis in 40.65% of cases, whereas it revealed an unexpected diagnosis in 22.5% of them. Syndromic diagnosis can orient the clinician toward the most probable kidney disease and guide any emergency treatment while awaiting RNB results. But it can never replace RNB which remains the gold standard.

## Introduction

L'apport de la ponction-biopsie rénale (PBR) dans le diagnostic, le choix thérapeutique et l’évaluation pronostique des maladies rénales est considérable en néphrologie clinique. Elle permet l'identification précise des lésions rénales et leur classification nosologique, l'appréciation des signes d'activité et de gravité, et l’évaluation de l'importance des lésions chroniques. Après une revue de la littérature, nous n'avons trouvé aucune étude marocaine ayant évalué la pratique et l'apport de cet examen indispensable dans les Services de néphrologie marocains. L'objectif de notre travail est d’étudier les indications de la PBR, déterminer la fréquence des maladies rénales identifiées par PBR dans notre région et de faire une confrontation entre les données clinico-biologiques et le diagnostic histologique.

## Méthodes

**Type d’étude**: Notre étude est rétrospective. Elle a été menée au sein du service de Néphrologie du Centre Hospitalier Universitaire (CHU) de Fès, entre Janvier 2009 et Décembre 2012.

**Patients:** Ils ont été sélectionnés à partir du registre d'anatomopathologie du service de Néphrologie. Nous avons inclus tous les patients ayant bénéficié d'une ponction biopsie de rein natif entre Janvier 2009 et Décembre 2012. Ont été exclus de l’étude les patients transplantés ayant bénéficié d'une biopsie du greffon rénal et ceux hospitalisés dans les autres services.

**Eléments recueillis:** Les données ont été recueillies à partir des dossiers médicaux des patients. Nous avons relevés les éléments suivants: aspects démographiques, cliniques, paracliniques des patients, le diagnostic initial suspecté par l’équipe de néphrologie et le diagnostic final retenu par l'anatomopathologiste. Les principaux syndromes ont été définis comme suit [1, 2]: Syndrome néphrotique (SN): protéinurie supérieure à 3 g/j et hypo-albuminémie inférieure à 30 g/l; Protéinurie non néphrotique isolée ou associée à une hématurie microscopique (PU/HU); Syndrome néphritique aigu: début brutal d'une insuffisance rénale aiguë (IRA) d'intensité variable, hypertension artérielle (HTA) et une protéinurie parfois de volume néphrotique; Syndrome de glomérulonéphrite rapidement progressive (GNRP): IRA d'aggravation rapide associée à une hématurie macroscopique inaugurale, l'absence habituelle d'HTA, et une protéinurie; Syndrome de néphropathie tubulo-interstitielle (SNTI): protéinurie en règle < 1 g/Jour sans HTA ni œdème, plus ou moins associés à l'hématurie et à l'IRA; Syndrome de néphropathie vasculaire (SNV): HTA au premier plan, l'absence d'anomalie majeure à l'examen du sédiment urinaire et la présence d'une insuffisance rénale souvent sévère et rapidement progressive; Insuffisance rénale aigue (IRA) isolée: élévation récente de la créatinine sérique, sans éléments en faveur de chronicité (absence d'anémie et d'hypocalcémie et des reins gardant une taille normale et une bonne différenciation à l’échographie rénale); Insuffisance rénale chronique inexpliquée: présence des critères en faveur du caractère chronique de la maladie rénale avec des reins de taille normale et gardant une bonne différenciation.

**Analyse statistique:** Elle a été effectuée grâce à la collaboration du Laboratoire d’épidémiologie, de recherche clinique et de santé communautaire de la Faculté de Médecine de Fès. Nous avons utilisé le logiciel Epi-info version 2000. Dans un premier temps, une analyse descriptive des caractéristiques démographiques, cliniques et biologiques des patients a été effectuée. Les résultats sont présentés sous forme de pourcentage et de moyennes ± Ecart type. Nous avons ensuite évalué le mode de présentation des différentes maladies rénales diagnostiquées par PBR et comparé le diagnostic suspecté cliniquement à celui retenu par l'anatomopathologiste.

## Résultats

**Figure 1:**
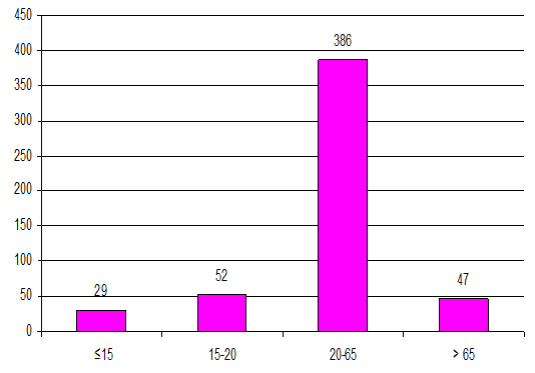
Répartition des biopsies rénales selon l’âge des patients

**Figure 2:**
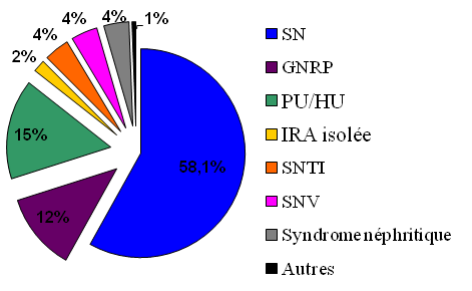
Diagnostics syndromiques chez nos patients au moment de la PBR

Durant une période de quatre ans, 522 PBR ont été réalisées chez 484 patients. En effet, 38 malades ont bénéficié (à des moments différents de leur suivi) de 2 biopsies pour: nombre insuffisant de glomérule sur 27 prélèvements et pour des raisons diagnostiques et thérapeutiques dans 11 cas. Nous avons exclu 8 biopsies devant le manque de renseignements cliniques sur les dossiers médicaux. Nous avons donc retenu 514 PBR. L’âge moyen de nos patients au moment de la PBR est de 39 ±17 ans, avec des extrêmes entre 3 et 82 ans. La grande majorité des patients (85,2%) est âgée entre 15 et 65 ans, avec une moyenne de 38,4 ± 13. Les patients âgés de plus de 65 ans représentent 9,4%. Leur âge moyen est de 71,6 ± 4,7. Les enfants (≤ 15 ans) représentent 5,6% de la population d’étude avec une moyenne d’âge de 10.7 ± 1,4 ans (Figure 1). La PBR a été réalisée chez 267 femmes et 247 hommes, soit un sex ration de 0,9. L'HTA est retrouvée chez presque la moitié des patients (43,3%) et la réduction de la diurèse chez seulement 8,6%. Le dosage pondéral de l'excrétion urinaire des protéines de 24 heures montre une protéinurie néphrotique chez 64,3% des patients. L'hématurie microscopique est retrouvée chez 65,4% d'entre eux. Nous avons noté la présence d'une insuffisance rénale au moment de la PBR chez 58,2% des malades. La PBR a été effectuée chez 29 patients diabétiques, soit 5,6% (Tableau 1). Le SN est le plus fréquent dans notre population d’étude (58,2%), tout âge confondu. La présence d'une PU/HU représente la deuxième indication de la PBR (16%), suivie par le syndrome de GNRP (12%) (Figure 2). L'analyse des données cliniques, biologiques et histologiques a permis d'orienter le diagnostic anatomoclinique définitif. Les néphropathies glomérulaires représentent 94,2% des maladies rénales diagnostiquées par PBR dans notre étude. Elles sont primitives chez 48,5% de nos patients: Lésion glomérulaire minime (LGM): 13,7%, Hyalinose segmentaire et focale (HSF): 12,1%, Glomérulonéphrite extra-membraneuse (GEM): 10%, Glomérulonéphrite aigue post-infectieuse (GNA): 4,4%, Glomérulonéphrite membrano-proliférative (GNMP): 3,2%, Néphropathie à IgA (N IgA): 3,2%, Glomérulonéphrite extra-capillaire (GNEC): 1,9%. Les néphropathies glomérulaires secondaires représentent 31,5% et elles sont réparties comme suit: Glomérulonéphrite lupique (GL): 17,5%, Amylose rénale: 8,4%, Néphropathie diabétique: 3,7%, Vascularite: 1,9%, Les Glomérulonéphrite chronique (GNC) sont diagnostiquées dans 14,2% des cas. La PBR a révélé une néphropathie tubulo-interstitielle chez 3,2% des malades, dont 1,6% de néphrite tubulo-interstitielle aigue (NTIA) et 1,3% de néphrite tubulo-interstitielle chronique (NTIC). La nécrose tubulaire aigue est retrouvée sur 0,3% des PBR. La Microangiopathie thrombotique est notée chez 2,2% des patients.

**Tableau 1:** Caractéristiques clinico-biologiques des patients au moment de la PBR

Caractéristiques	Valeurs
Hypertension artérielle (%)	43,3
Protéinurie (g/24 heures;%)	
<0,3	4,7
0,3- 3	31
>3	64,3
Hématurie	65,4
Oligo-anurie	8,6
Insuffisance rénale	58,2

Dans le Tableau 2, sont présentées les causes de SN. La LGM en est la première chez les enfants. Chez les adultes, c'est la GEM qui vient au premier rang suivie de très près par la GL et l'amylose. Le SN révèle le plus souvent une GNC chez les sujets âgés. Nous avons diagnostiqué une LGM, une GL et une N IgA chez les 3 enfants ayant bénéficié d'une PBR pour une PU /HU. Chez les adultes, la GL prédomine dans cette catégorie syndromique. La PBR a été réalisée chez seulement 2 patients âgés pour PU/HU et a révélé une HSF et une GNC (Tableau 3). La GNC est la néphropathie la plus retrouvée chez les patients adultes présentant une GNRP, suivie par la vascularite (15,2%). La GNEC est retrouvée chez 2 adultes seulement (4,2%). Chez les personnes âgées, la GNMP prend le dessus (Tableau 4). Le syndrome néphritique a motivé la réalisation d'une PBR chez 16 patients adultes, la GNA en est la principale étiologie (47%). La GNC est retrouvée dans 3 cas parmi les 16 (18,7%) et la GL dans 2 (12,5%). Une NTIA est retrouvée chez le seul sujet âgé (67 ans) porteur de syndrome néphritique. Le SNTI est noté chez 13 adultes et est responsable d'une NTIA chez 38,5% d'entre eux et d'une NTIC chez 7,7%. Il est lié à une GNC chez 30,7%. La PBR a objectivé 2 cas de NTIC, une vascularite et une GNC chez les 4 patients âgés présentant un SNTI. Nous avons réalisé la PBR chez 13 adultes présentant un SNV, celle-ci a objectivé 3 cas de néphro-angiosclérose maligne (NASM), 3 GNC, une amylose, une LGM et une HSF (2 cas chacune). Un seul malade présente une micro-angiopathie thrombotique. Tandis que chez les sujets âgés, le SNV est due à l'amylose et à la GNC (1 cas chacun). Un seul enfant et un seul patient âgé de 67 ans ont bénéficié de la PBR devant la présence d'une IRA isolée, celle-ci a révélé une N IgA chez le premier et une vascularite chez le deuxième. Dans notre étude, le mode de présentation clinique des maladies rénales varie selon l’âge. L'amylose rénale est révélée principalement par un SN chez l'adulte (74%) et chez les sujets âgés (2 cas sur 3). Aucun cas n'est noté chez les enfants. La GNEC est retrouvée chez un seul enfant présentant un SN. Chez les adultes, elle s'est manifestée principalement par un SN (60%), suivi par une GNRP (40%). Aucun cas de GNRP n'est noté chez les patients âgés. La vascularite s'est présentée principalement par un syndrome de GNRP (77,7%) chez les adultes. Seulement 3 cas de vascularite sont diagnostiqués chez les personnes âgées, révélés chacun par une GNRP, une IRA isolée et un SNTI. La GEM se manifeste quasi-exclusivement par un SN dans toutes les tranches d’âge (Tableau 5). Le SN est également le mode de présentation le plus fréquent de la HSF, il est exclusif chez les enfants et les sujets âgés et est retrouvé chez 72,2% des adultes, suivi par la PU/HU (Tableau 6). La LGM se manifeste exclusivement par un SN chez les enfants. Chez les adultes, le SN est retrouvé chez 70,5% des cas, suivi par une PU/HU (14,7%) et une GNRP (8,8%). Chez les sujets âgés également, le SN est le mode de révélation le plus fréquent (75%), suivi par la GNRP (Tableau 7). Les modes de présentation de la N IgA varient en fonction de l’âge. Il s'agit, chez les enfants, de SN, de PU/HU et d'IRA isolée (33% chacun). Chez les adultes, la PU/HU est le mode de présentation le plus fréquent (83,3%) suivi par le SN. Un seul cas de N IgA est diagnostiqué chez un sujet âgé de 68 ans et il a été révélé par un SN. La GNA post-infectieuse se présente chez les adultes principalement par un syndrome néphritique (46,7%) et SN (33,3%). Nous avons retenu le diagnostic de GNA chez une seule personne âgée, elle s'est présentée sous forme de SN. Aucun cas n'est retrouvé chez les enfants. Le SN est le mode de présentation le plus fréquent de la GNMP dans toutes les tranches d’âge (100% chez les enfants, 66,7% chez les adultes et 50% les sujets âgés). Une PU/HU est retrouvée chez 16,7% des adultes. La GNRP chez 50% des sujets âgés. La GL se présente chez les adultes par un SN (52%), une PU/HU (38,9%), syndrome néphritique et GNRP (3,7% chacun) et IRA isolée (1,7%). Deux cas sont retrouvés chez les enfants et sont révélés par une PU/HU. Un seul patient âgé de plus de 65 ans, présente une GL qui s'est manifestée par un SN. La biopsie rénale a objectivé une GNC chez 72 patients (1 enfant, 62 adultes et 9 sujets âgés). Elle a été motivée par un SN chez l'enfant, par une GNRP (40,3%) suivie par un SN (33,8%), un SNTI (7%), et un syndrome néphritique (5%) chez les adultes. Chez les sujets âgés le SN prédomine avec 55,5%, le SNTI est présent chez 2 patients (22,2%), la GNRP et le SNV chez 11,1% chacun. La néphro-angiosclérose maligne est retrouvée chez 5 patients adultes, chez qui elle se manifeste par un SNV dans 75% des cas et une GNRP dans 25%. La PBR a confirmé le premier diagnostic suspecté cliniquement dans 40,65 des cas, elle était en faveur du diagnostic différentiel dans 36,86, alors qu'elle a révélé un nouveau diagnostic inattendu dans 22,5%.

**Tableau 2:** Répartition des néphropathies prouvées par PBR en cas de syndrome néphrotique

Diagnostic histologique	Enfantsn = 10; (%)	Adultes n = 212; (%)	Agés n = 25; (%)
LGM	30	11,3	12
HSF	10	7,5	12
GEM	0	14	12
LGM ou HSF	0	8,5	0
GNMP	0	1,9	8
Non classée	40	17,4	12
GNEC	10	1,4	0
GNA	0	2,4	4
N IgA	10	0,5	4
GL	0	13,2	4
Amylose	0	11	8
Vascularite	0	0,9	0
GNC	0	9	20
NTA	0	0,5	0
NTIA	0	0,5	0
Autre	0	0,5	4

**Tableau 3:** Répartition des néphropathies prouvées par PBR en cas de protéinurie/ hématurie microscopique

Diagnostic histologique	Enfants n = 3; (%)	Adultes n = 62; (%)	Agés n = 2; (%)
LGM	33,33	8	0
HSF	0	6,4	50
GEM	0	4,8	0
LGM ou HSF	0	11,3	0
GNMP	0	1,6	0
Non classée	0	14,5	0
GNEC	0	0	0
GNA	0	1,6	0
N IgA	33,33	8	0
GL	33,33	33,9	0
Amylose	0	1,6	0

**Tableau 4:** Fréquence des différentes néphropathies prouvées par biopsie en cas de GNRP

Diagnostic histologique	Adultesn = 47; (%)	Agés n = 5; (%)
LGM	6,4	20
HSF	2,1	0
GEM	0	0
LGM ou HSF	0	0
GNMP	0	40
Non classée	4,2	0
GNEC	4,2	0
GNA	4,2	0
N IgA	0	0
GL	4,2	0
Amylose	4,2	0
Vascularite	15,2	20
GNC	49	20
NTA	0	0
NTIA	0	0
MAT et NASM	2,1	0
NTIC	4,2	0

**Tableau 5:** Modes de présentation clinique de la GEM selon l’âge

Diagnostics syndromiques	Enfants n = 1; (%)	Adultes n = 39; (%)	Agés n = 4; (%)
SN	1	85,3	100
PU/HU	0	8,8	0
GNRP	0	0	0
Syndrome néphritique	0	2,9	0
IRA isolée	0	2,9	0

**Tableau 6:** Modes de présentation clinique de la HSF selon l’âge

Diagnostics syndromiques	Enfants n = 2; (%)	Adultesn = 29; (%)	Agés n = 3; (%)
SN	100	72,7	100
PU/HU	0	18,3	0
GNRP	0	4,5	0
Syndrome néphritique	0	0	0
IRA isolée	0	0	0
SNV		4,5	0

**Tableau 7:** Modes de présentation clinique de la LGM selon l’âge

Diagnostics syndromiques	Enfantsn = 3; (%)	Adultesn = 34; (%)	Agés n = 4; (%)
SN	100	70,5	75
PU/HU	0	14,7	0
GNRP	0	8,8	25
Syndrome néphritique	0	0	0
IRA isolée	0	0	0
SNV	0	6	0

## Discussion

La pratique de la PBR en néphrologie clinique manque considérablement dans de nombreux pays en voie de développement et particulièrement en Afrique [3]. Le service de Néphrologie du CHU de Fès est un parmi les quatre qui pratiquent la PBR au Maroc. Et selon la répartition administrative du pays, le CHU de Fès draine toute la population de la région de Fès-Boulemane. Ainsi, les résultats de notre étude s’étalant sur quatre ans pourraient être représentatifs des maladies rénales diagnostiquées par PBR dans cette région. Le SN est le mode de présentation le plus fréquent des maladies rénales chez nos patients de tout âge. Il représente ainsi la première indication de la PBR avec une fréquence de 58,1%. Ce résultat est comparable à celui d'une étude menée en Afrique du Sud [4] avec une fréquence de 52,5% et à ceux d'autres études réalisées dans d'autres régions du monde [5–7]. Chez l'ensemble de nos patients, les premières causes de SN sont la GEM, la LGM et la HSF. Ceci a été retrouvé également dans d'autres études [8]. La PBR n'est pas systématique en cas de SN chez l'enfant, dans notre série, 6 en ont bénéficié devant la présence d'un SN impur. La LGM en est la principale cause (30%), suivie par la HSF (10%). Ceci est en concordance avec la plupart des études. Ainsi, dans le rapport du Registre Italien des biopsies rénales, le SN chez l'enfant est attribué principalement à la LGM (34,5%) et à la HSF (16,9%) [9]. De même, dans la grande série Coréenne ayant inclut 426 enfants présentant un SN (71,4% de LGM et 12,2% de HSF) [10]. Chez les personnes âgées, le SN est lié principalement à la GNC suivie et à la même proportion par la LGM, la HSF et la GEM. Cette fréquence des GNC pourrait être expliquée par le retard de réalisation de la PBR, lié le plus souvent à la consultation tardive chez cette population, ainsi qu’à l'absence de bilan antérieur pouvant orienter vers le caractère aigu ou chronique de la maladie rénale. La présence d'une PU/HU est la deuxième indication de la PBR dans notre série (16%). C'est le cas également dans une étude Tchèque, mais avec une fréquence plus élevée (36,2%) [11]. Les 3 glomérulopathies identifiées chez les 3 enfants ayant bénéficié d'une PBR pour PU/HU sont: la LGM, la N IgA et la GL. Ces résultats sont différents de ceux rapportés par le Registre Italien qui inclut 135 cas (30,4% N IgA et GNMP 5,2%) [12] et par la Série Japonaise incluant 54 patients (54% N IgA, GEM 5,5%) [13]. Ces discordances pourraient être liées au petit nombre d'enfants dans notre étude, mais elles pourraient refléter notre approche prudente envers les biopsies rénales chez les enfants dans notre service (2 enfants parmi les 3 présentaient des signes extra-rénaux au moment de le PBR). La GNA se manifeste le plus souvent chez l'enfant par un syndrome néphritique, son évolution est souvent favorable et nécessite rarement la réalisation de la PBR. Dans notre série, aucun enfant n'a bénéficié de la PBR pour syndrome néphritique. Dans une étude Espagnole, ce syndrome était principalement causé par la N IgA et la GNA chez la moitié des enfants et par la GL et la vascularite chez les adultes. Tandis que la GNA post-infectieuse représente la première cause de syndrome néphritique chez les sujets adultes dans notre série. Cette discordance pourrait être liée à la persistance des maladies infectieuses en rapport avec le niveau de santé et le niveau socio-économique dans notre pays.

L'IRA isolée est le syndrome clinique le moins fréquent chez les patients ayant bénéficié de la PBR dans notre étude. En effet, dans la grande majorité des cas, la cause de l'IRA isolée est évidente cliniquement sans avoir recours à la PBR et sa correction a permis une correction rapide de la fonction rénale. La même constatation est notée dans l’étude de Naumovic [5]. Dans notre étude, la PBR réalisée chez les patients adultes présentant une GNRP a révélé une GNC dans 49% des cas, une vascularite dans 15,2%, une LGM dans 6,4% et une GNEC dans 4,2%. Chez les sujets âgés, les causes de la GNRP sont très variées et la vascularite en représente 20%. La vascularite et la GNEC sont les causes principales retrouvées chez les adultes et les sujets âgés dans la littérature [12, 14]. Parmi les groupes histologiques déterminés par la PBR, les néphropathies glomérulaires sont majoritaires avec une fréquence de 94,1%. Elles représentent la première cause des maladies rénales prouvées par biopsie dans de nombreux registres [15, 16]. Les néphropathies glomérulaires secondaires sont plus fréquentes dans notre étude en comparaison avec plusieurs séries européennes [8, 11, 17]. Ceci pourrait être expliqué par une prévalence plus importante de la GL qui représente la première étiologie des glomérulopathies secondaires dans notre série. Chez les adultes, la GL se présente principalement par un SN et une PU/HU. Alors que le syndrome néphritique, la GNRP et l'IRA isolée sont rares. Ces résultats pourraient signaler en effet, la réalisation précoce de la PBR chez nos jeunes patients lupiques dès les premiers signes d'atteinte rénale. Dans notre série, la GL est très rare chez les enfants et les personnes âgées. La N IgA est la première cause des glomérulopathies primitives dans le monde entier, ceci a été confirmé par plusieurs études en Italie [12, 18]. Alors qu'elle représente, la deuxième glomérulopathie primitive la moins fréquente dans notre série. Ceci pourrait être dû à notre stratégie de ne pas réaliser la PBR systématiquement en cas de forte suspicion de N IgA et en absence de signes de gravité (hématurie macroscopique récidivante, hématurie microscopique isolée ou associée à une protéinurie modérée et en absence d'insuffisance rénale et de signes extra-rénaux). Le SN est le mode de présentation le plus fréquent de la GNMP à tous les âges. Ces résultats sont comparables à ceux obtenus dans la grande série de GNMP publiée dans les années 1970 et 1980 par une étude Espagnole [19]. D'un autre côté, une diminution de la fréquence de la GNMP est observée au fil des années dans les pays développés [19]. Nous n'avons pas la possibilité de vérifier cette donnée dans notre contexte Marocain, vu le manque d’études. Néanmoins, nous notons que la GNMP est la glomérulopathie primitive la moins fréquente dans notre série (3,4%). Le premier diagnostic étiologique suspecté sur les données cliniques et biologiques dans notre étude a été retenu après la confrontation aux résultats histologiques dans 40,65% des cas. Notre raisonnement diagnostique et par conséquent thérapeutique a été complètement modifié par le résultat histologique dans 22,49% des cas. Ces données soulignent l'intérêt de la PBR dans la prise en charge des maladies rénales. Le diagnostic histologique est d'une grande importance en néphrologie clinique. Il permet, en effet, de guider le traitement approprié, en particulier en cas de vascularite rénale et de GNEC qui représentent les grandes urgences néphrologiques diagnostiques et thérapeutiques. La présente étude fournit une description générale des maladies rénales prouvées par PBR dans la région de Fès-Boulemane. Toutefois, ces résultats doivent être interprétés avec prudence vu le caractère rétrospectif et le petit échantillon de notre série, en comparaison avec la littérature. Elle pourrait représenter une première étape vers une étude multicentrique qui permettrait de comparer les caractéristiques cliniques et histologiques des maladies rénales entre les différentes régions du Maroc, afin de déterminer la part des facteurs environnementaux et raciaux dans la survenue de certaines maladies rénales et d'orienter les protocoles diagnostiques et thérapeutiques

## Conclusion

Le SN représente le premier mode de présentation clinique des maladies rénales, la LGM, la HSF et la GEM en sont les premières causes. Le diagnostic syndromique permet au clinicien d'identifier la maladie rénale la plus probable et de guider les thérapeutiques urgentes en attendant les résultats de la PBR. Cependant, cette approche ne doit pas être considérée comme une alternative à la PBR qui reste le gold standard du diagnostic de la maladie rénale.

### Etat des connaissances actuelle sur le sujet

- La PBR a un intérêt diagnostique, pronostique et thérapeutique en néphrologie clinique.

### Contribution de notre étude à la connaissance

- Première étude marocaine sur la fréquence des maladies rénales et leurs répartitions;
- Une bonne analyse de la sémiologie clinico-biologique permet d'orienter le diagnostic, de guider le bilan étiologique et même d'instaurer un traitement en cas d'urgence, en attendant les résultats de la PBR.
